# Creatininium 2-chloro­acetate

**DOI:** 10.1107/S1600536812012068

**Published:** 2012-04-04

**Authors:** A. Jahubar Ali, S. Athimoolam, S. Asath Bahadur

**Affiliations:** a Department of Science and Humanities, National College of Engineering, Maruthakulam, Tirunelveli 627 151, India; b Department of Physics, University College of Engineering Nagercoil, Anna University of Technology, Tirunelveli, Nagercoil 629 004, India; c Department of Physics, Kalasalingam University, Anand Nagar, Krishnan Koil 626 190, India

## Abstract

In the title compound (systematic name: 2-amino-1-methyl-4-oxo-4,5-dihydro-1*H*-imidazol-3-ium 2-chloro­acetate), C_4_H_8_N_3_O^+^·C_2_H_2_ClO_2_^−^, the mol­ecular aggregations are stabil­ized through classical (N—H⋯O) and non-classical (C—H⋯O and C—H⋯N) hydrogen-bonding inter­actions. The cations are linked to the anions, forming ion pairs through two N—H⋯O bonds that produce characteristic *R*_2_^2^(8) ring motifs. These cation–anion pairs are connected through another N—H⋯O hydrogen bond, leading to an *R*_4_^2^(8) ring motif. Further weak C—H⋯N inter­actions link the mol­ecules along the *a* axis, while other C—H⋯O inter­actions generate zigzag chains extending along *b*.

## Related literature

For related structures, see: Ali *et al.* (2011*a* ▶,*b* ▶); Bahadur, Kannan *et al.* (2007 ▶); Bahadur, Sivapragasam *et al.* (2007 ▶); Bahadur, Rajalakshmi *et al.* (2007 ▶). For hydrogen-bond motifs, see: Bernstein *et al.* (1995 ▶). For the biological importance of creatinine, see: Madaras & Buck (1996 ▶); Sharma *et al.* (2004 ▶); Narayanan & Appleton (1980 ▶).
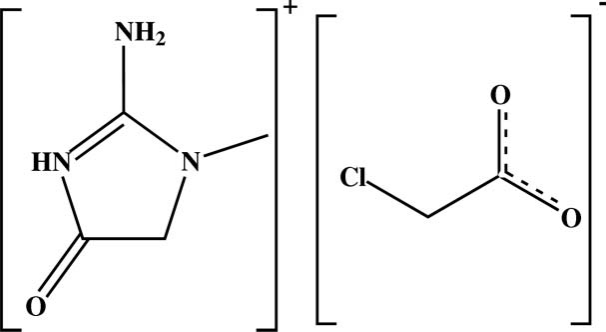


## Experimental

### 

#### Crystal data

C_4_H_8_N_3_O^+^·C_2_H_2_ClO_2_^−^
*M* *_r_* = 207.62
Monoclinic, 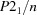

*a* = 5.7993 (8) Å
*b* = 13.934 (2) Å
*c* = 11.2205 (16) Å
β = 95.326 (2)°
*V* = 902.8 (2) Å^3^
*Z* = 4
Mo *K*α radiation
μ = 0.40 mm^−1^
*T* = 294 K
0.24 × 0.22 × 0.19 mm

#### Data collection

Bruker SMART APEX CCD area-detector diffractometer
8205 measured reflections
1587 independent reflections
1472 reflections with *I* > 2σ(*I*)
*R*_int_ = 0.031

#### Refinement

*R*[*F* ^2^ > 2σ(*F* ^2^)] = 0.041
*wR*(*F* ^2^) = 0.117
*S* = 1.05
1587 reflections
131 parameters
H atoms treated by a mixture of independent and constrained refinement
Δρ_max_ = 0.33 e Å^−3^
Δρ_min_ = −0.27 e Å^−3^

### 

Data collection: *SMART* (Bruker, 2001 ▶); cell refinement: *SAINT* (Bruker, 2001 ▶); data reduction: *SAINT*; program(s) used to solve structure: *SHELXTL/PC* (Sheldrick, 2008 ▶); program(s) used to refine structure: *SHELXTL/PC*; molecular graphics: *PLATON* (Spek, 2009 ▶); software used to prepare material for publication: *SHELXTL/PC*.

## Supplementary Material

Crystal structure: contains datablock(s) global, I. DOI: 10.1107/S1600536812012068/sj5215sup1.cif

Structure factors: contains datablock(s) I. DOI: 10.1107/S1600536812012068/sj5215Isup2.hkl

Supplementary material file. DOI: 10.1107/S1600536812012068/sj5215Isup3.cml

Additional supplementary materials:  crystallographic information; 3D view; checkCIF report

## Figures and Tables

**Table 1:** Hydrogen-bond geometry (Å, °)

*D*—H⋯*A*	*D*—H	H⋯*A*	*D*⋯*A*	*D*—H⋯*A*
N15—H1*N*⋯O22^i^	0.81 (3)	2.02 (3)	2.762 (2)	152 (2)
N15—H2*N*⋯O22	0.94 (3)	1.82 (3)	2.758 (2)	179 (2)
N14—H14*N*⋯O21	0.86 (3)	1.83 (3)	2.686 (2)	173 (2)
C11—H11*A*⋯O13^ii^	0.96	2.46	3.305 (3)	147
C11—H11*B*⋯O13^iii^	0.96	2.55	3.448 (3)	156
C12—H12*A*⋯N15^iv^	0.97	2.78	3.695 (3)	157
C12—H12*B*⋯O21^iii^	0.97	2.36	3.208 (2)	146

Symmetry codes: (i) 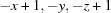; (ii) 
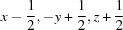
; (iii) 
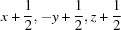
; (iv) 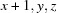.

**Fig. 1.**
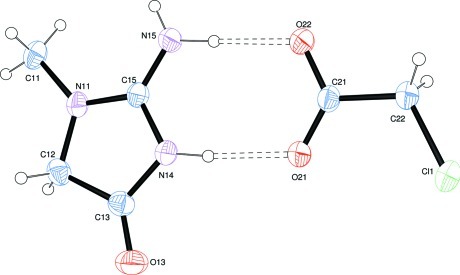
The asymmetric unit of the title compound (I) with the numbering scheme for the atoms and 50% probability displacement ellipsoids. H bonds are drawn as dashed lines.

**Fig. 2.**
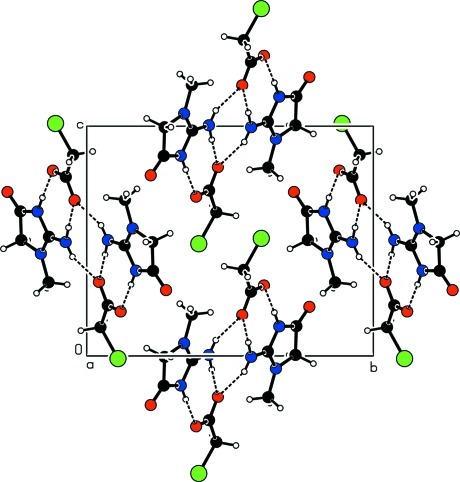
Packing diagram of the molecules viewed down the b-axis. H atoms not involved in the H-bonds (dashed lines) are omitted for clarity.

## supplementary crystallographic information

### Comment

Creatinine, a nitrogenous organic acid, is found in the muscle tissue of vertebrates mainly in the form of phosphocreatine and supplies energy for muscle contraction. Also, it is a blood metabolite of considerable importance in clinical chemistry, particularly as an indicator of renal function. It has been proven that determination of creatinine is more valuable for the detection of renal dysfunction than that of urea (Sharma *et al.*, 2004). In renal physiology, creatinine clearance rate, CCr, (Madaras & Buck, 1996) is the volume of blood plasma that is cleared of creatinine per unit time. Clinically, creatinine clearance is a useful measure for estimating the glomerular filtration rate (GFR) of the kidneys. An abnormal level of creatinine in biological fluids is an indicator of various disease states (Narayanan & Appleton, 1980). Also, the effective protonation site on the creatinine molecule (N atoms) can form intermolecular interactions such as hydrogen bonds that play an essential role in the formation of supramolecular systems. As we have stated in our previous papers, we are interested on the the specificity of recognition between inorganic / organic acids and the cretinine molecule. Hence, the title compound is reported here.

The asymmetric unit of the title compound, (I), contains one protonated creatinine molecule as the creatininium cation and one deprotonated monochloroacetic acid as the monochloroacetate anion (Fig.1). Protonation of the N site of the cation is evident from C—N bond distances and the C—N—C bond angle. Other bond distances and angles are comparable with those found in creatininium hydrogen maleate (Ali *et al.*, 2011*a*), creatininium cinnamate (Ali *et al.*, 2011*b*), creatininium hydrogen oxalate monohydrate (Bahadur, Kannan *et al.*, 2007), creatininium benzoate (Bahadur, Sivapragasam *et al.*, 2007) and bis(creatininium) sulfate (Bahadur, Rajalakshmi *et al.*, 2007). The deprotonation on the –COOH groups of the monochloroacetic acid is confirmed from the –COO^-^ bond geometry. The plane of the five membered ring in the cation and that of the carboxylate group of the anion are oriented at an angle of 9.5 (1)°.

In the crystal structure, molecular aggregations are stabilized through classical (N—H···O) and non-classical (C—H···O and C—H···N) hydrogen bonding interactions (Table 1). Cations are linked to anions forming ion pairs through two N—H···O bonds that produce characterestic *R*_2_^2^(8) ring motifs (Bernstein *et al.*, 1995). This type of ring motif is observed in most structures of creatinine salts of inorganic/organic acids, especially when carboxylate anions are present (Fig. 2). These cation-anion pairs are connected through another N—H···O hydrogen bond leading to an *R*_4_^2^(8) ring motif around the inversion centres of the unit cell. These centrosymmetric ring motifs are almost planar and oriented with an angle of 78.1 (1)° to each other and lie in the (131) and (131) planes respectively. leading to strong diffraction peaks for the planes. These molecular aggregates are further connected through three C—H···O and one C—H···N interactions. The C—H···N contacts link the molecules along the *a* axis. Further, other C—H···O interactions generate zigzag chains extending along *b*. Notably, the electronegative Cl atoms are not involved in any classical or non-classical hydrogen bonding interactions.

### Experimental

The title compound was crystallized from an aqueous mixture containing creatinine and monochloroacetic acid in a 1:1 stoichiometric ratio at room temperature by the slow evaporation technique.

### Refinement

H atoms bound to N and involved in hydrogen bonds were located from a difference Fourier map and refined isotropically. Other H atoms except were positioned geometrically and refined using a riding model, with C—H = 0.93 (–CH) and 0.96 Å (–CH_3_) and *U*_iso_(H) = 1.2–1.5 *U*_eq_ (parent atom).

### Crystal data

**Table:**

C_4_H_8_N_3_O^+^·C_2_H_2_ClO_2_^−^	*F*(000) = 432
*M**_r_* = 207.62	*D*_x_ = 1.527 Mg m^−^^3^
Monoclinic, *P*2_1_/*n*	Mo *K*α radiation, λ = 0.71073 Å
Hall symbol: -P 2yn	Cell parameters from 2516 reflections
*a* = 5.7993 (8) Å	θ = 2.2–23.4°
*b* = 13.934 (2) Å	µ = 0.40 mm^−^^1^
*c* = 11.2205 (16) Å	*T* = 294 K
β = 95.326 (2)°	Block, colourless
*V* = 902.8 (2) Å^3^	0.24 × 0.22 × 0.19 mm
*Z* = 4	

### Data collection

**Table:**

Bruker SMART APEX CCD area-detector diffractometer	1472 reflections with *I* > 2σ(*I*)
Radiation source: fine-focus sealed tube	*R*_int_ = 0.031
Graphite monochromator	θ_max_ = 25.0°, θ_min_ = 2.3°
ω scans	*h* = −6→6
8205 measured reflections	*k* = −16→16
1587 independent reflections	*l* = −13→13

### Refinement

**Table:**

Refinement on *F*^2^	Primary atom site location: structure-invariant direct methods
Least-squares matrix: full	Secondary atom site location: difference Fourier map
*R*[*F*^2^ > 2σ(*F*^2^)] = 0.041	Hydrogen site location: inferred from neighbouring sites
*wR*(*F*^2^) = 0.117	H atoms treated by a mixture of independent and constrained refinement
*S* = 1.05	*w* = 1/[σ^2^(*F*_o_^2^) + (0.0666*P*)^2^ + 0.3396*P*] where *P* = (*F*_o_^2^ + 2*F*_c_^2^)/3
1587 reflections	(Δ/σ)_max_ < 0.001
131 parameters	Δρ_max_ = 0.33 e Å^−^^3^
0 restraints	Δρ_min_ = −0.27 e Å^−^^3^

### Special details

**Table:**

Geometry. All e.s.d.'s (except the e.s.d. in the dihedral angle between two l.s. planes) are estimated using the full covariance matrix. The cell e.s.d.'s are taken into account individually in the estimation of e.s.d.'s in distances, angles and torsion angles; correlations between e.s.d.'s in cell parameters are only used when they are defined by crystal symmetry. An approximate (isotropic) treatment of cell e.s.d.'s is used for estimating e.s.d.'s involving l.s. planes.
Refinement. Refinement of *F*^2^ against ALL reflections. The weighted *R*-factor *wR* and goodness of fit *S* are based on *F*^2^, conventional *R*-factors *R* are based on *F*, with *F* set to zero for negative *F*^2^. The threshold expression of *F*^2^ > σ(*F*^2^) is used only for calculating *R*-factors(gt) *etc*. and is not relevant to the choice of reflections for refinement. *R*-factors based on *F*^2^ are statistically about twice as large as those based on *F*, and *R*- factors based on ALL data will be even larger.

### Fractional atomic coordinates and isotropic or equivalent isotropic displacement parameters (Å^2^)

**Table:**

	*x*	*y*	*z*	*U*_iso_*/*U*_eq_	
N11	1.0950 (2)	0.16015 (11)	0.55491 (13)	0.0435 (4)	
C11	1.1347 (4)	0.13325 (16)	0.67962 (17)	0.0517 (5)	
H11A	1.0491	0.1753	0.7270	0.078*	
H11B	1.2969	0.1383	0.7052	0.078*	
H11C	1.0845	0.0683	0.6895	0.078*	
C12	1.2339 (3)	0.22831 (14)	0.49571 (17)	0.0474 (5)	
H12A	1.3926	0.2064	0.4957	0.057*	
H12B	1.2328	0.2908	0.5336	0.057*	
C13	1.1149 (3)	0.23140 (13)	0.37071 (18)	0.0471 (5)	
O13	1.1682 (3)	0.27677 (12)	0.28629 (14)	0.0668 (5)	
N14	0.9267 (3)	0.17187 (11)	0.37131 (14)	0.0434 (4)	
C15	0.9179 (3)	0.13198 (12)	0.48112 (15)	0.0390 (4)	
N15	0.7527 (3)	0.07372 (13)	0.50395 (16)	0.0482 (4)	
H1N	0.741 (4)	0.0498 (17)	0.569 (2)	0.063 (7)*	
H2N	0.636 (4)	0.0637 (17)	0.441 (2)	0.062 (6)*	
H14N	0.835 (4)	0.1546 (17)	0.311 (2)	0.062 (7)*	
C21	0.4400 (3)	0.07309 (13)	0.22026 (16)	0.0434 (4)	
C22	0.2464 (4)	0.04780 (17)	0.12518 (18)	0.0561 (5)	
H22A	0.0995	0.0614	0.1564	0.067*	
H22B	0.2520	−0.0206	0.1099	0.067*	
O21	0.6111 (2)	0.11830 (11)	0.19341 (13)	0.0578 (4)	
O22	0.4072 (2)	0.04254 (11)	0.32210 (12)	0.0547 (4)	
Cl1	0.25524 (10)	0.10901 (5)	−0.01176 (5)	0.0725 (3)	

### Atomic displacement parameters (Å^2^)

**Table:**

	*U*^11^	*U*^22^	*U*^33^	*U*^12^	*U*^13^	*U*^23^
N11	0.0406 (8)	0.0488 (8)	0.0392 (8)	−0.0010 (6)	−0.0055 (6)	−0.0015 (6)
C11	0.0518 (11)	0.0622 (12)	0.0390 (10)	0.0059 (9)	−0.0074 (8)	−0.0025 (9)
C12	0.0363 (9)	0.0517 (11)	0.0536 (11)	−0.0030 (7)	0.0016 (8)	−0.0055 (8)
C13	0.0405 (9)	0.0501 (10)	0.0510 (11)	−0.0005 (8)	0.0067 (8)	0.0015 (8)
O13	0.0589 (9)	0.0813 (11)	0.0612 (10)	−0.0129 (7)	0.0108 (7)	0.0176 (8)
N14	0.0409 (8)	0.0507 (9)	0.0373 (8)	−0.0039 (6)	−0.0028 (6)	0.0016 (6)
C15	0.0382 (9)	0.0401 (8)	0.0379 (9)	0.0037 (7)	−0.0009 (7)	−0.0010 (7)
N15	0.0462 (10)	0.0556 (10)	0.0414 (9)	−0.0097 (7)	−0.0043 (8)	0.0082 (7)
C21	0.0448 (10)	0.0456 (9)	0.0380 (9)	−0.0027 (7)	−0.0056 (8)	0.0014 (7)
C22	0.0539 (11)	0.0694 (13)	0.0427 (10)	−0.0161 (10)	−0.0087 (9)	0.0053 (9)
O21	0.0544 (8)	0.0748 (10)	0.0418 (8)	−0.0223 (7)	−0.0081 (6)	0.0098 (6)
O22	0.0545 (8)	0.0661 (9)	0.0413 (8)	−0.0140 (7)	−0.0068 (6)	0.0123 (6)
Cl1	0.0710 (4)	0.0998 (5)	0.0426 (3)	−0.0207 (3)	−0.0164 (3)	0.0133 (3)

### Geometric parameters (Å, º)

**Table:**

N11—C15	1.318 (2)	N14—C15	1.357 (2)
N11—C12	1.446 (2)	N14—H14N	0.86 (3)
N11—C11	1.446 (2)	C15—N15	1.299 (2)
C11—H11A	0.9600	N15—H1N	0.81 (3)
C11—H11B	0.9600	N15—H2N	0.94 (3)
C11—H11C	0.9600	C21—O21	1.236 (2)
C12—C13	1.505 (3)	C21—O22	1.251 (2)
C12—H12A	0.9700	C21—C22	1.517 (2)
C12—H12B	0.9700	C22—Cl1	1.762 (2)
C13—O13	1.203 (2)	C22—H22A	0.9700
C13—N14	1.371 (2)	C22—H22B	0.9700
			
C15—N11—C12	109.98 (15)	C15—N14—C13	110.45 (16)
C15—N11—C11	125.12 (16)	C15—N14—H14N	122.0 (16)
C12—N11—C11	124.71 (15)	C13—N14—H14N	127.1 (16)
N11—C11—H11A	109.5	N15—C15—N11	127.46 (17)
N11—C11—H11B	109.5	N15—C15—N14	121.71 (16)
H11A—C11—H11B	109.5	N11—C15—N14	110.83 (16)
N11—C11—H11C	109.5	C15—N15—H1N	124.1 (18)
H11A—C11—H11C	109.5	C15—N15—H2N	116.0 (14)
H11B—C11—H11C	109.5	H1N—N15—H2N	120 (2)
N11—C12—C13	102.72 (14)	O21—C21—O22	126.16 (17)
N11—C12—H12A	111.2	O21—C21—C22	120.37 (16)
C13—C12—H12A	111.2	O22—C21—C22	113.46 (16)
N11—C12—H12B	111.2	C21—C22—Cl1	114.92 (14)
C13—C12—H12B	111.2	C21—C22—H22A	108.5
H12A—C12—H12B	109.1	Cl1—C22—H22A	108.5
O13—C13—N14	125.77 (19)	C21—C22—H22B	108.5
O13—C13—C12	128.30 (18)	Cl1—C22—H22B	108.5
N14—C13—C12	105.92 (16)	H22A—C22—H22B	107.5
			
C15—N11—C12—C13	−3.13 (19)	C11—N11—C15—N15	−2.4 (3)
C11—N11—C12—C13	−178.40 (16)	C12—N11—C15—N14	2.9 (2)
N11—C12—C13—O13	−178.5 (2)	C11—N11—C15—N14	178.17 (16)
N11—C12—C13—N14	2.23 (19)	C13—N14—C15—N15	179.15 (17)
O13—C13—N14—C15	−179.97 (19)	C13—N14—C15—N11	−1.4 (2)
C12—C13—N14—C15	−0.7 (2)	O21—C21—C22—Cl1	−13.4 (3)
C12—N11—C15—N15	−177.63 (18)	O22—C21—C22—Cl1	167.75 (15)

### Hydrogen-bond geometry (Å, º)

**Table:**

*D*—H···*A*	*D*—H	H···*A*	*D*···*A*	*D*—H···*A*
N15—H1*N*···O22^i^	0.81 (3)	2.02 (3)	2.762 (2)	152 (2)
N15—H2*N*···O22	0.94 (3)	1.82 (3)	2.758 (2)	179 (2)
N14—H14*N*···O21	0.86 (3)	1.83 (3)	2.686 (2)	173 (2)
C11—H11*A*···O13^ii^	0.96	2.46	3.305 (3)	147
C11—H11*B*···O13^iii^	0.96	2.55	3.448 (3)	156
C12—H12*A*···N15^iv^	0.97	2.78	3.695 (3)	157
C12—H12*B*···O21^iii^	0.97	2.36	3.208 (2)	146

Symmetry codes: (i) −*x*+1, −*y*, −*z*+1; (ii) *x*−1/2, −*y*+1/2, *z*+1/2; (iii) *x*+1/2, −*y*+1/2, *z*+1/2; (iv) *x*+1, *y*, *z*.
